# Hepatotoxicidad colestásica asociada a tratamiento con enoxaparina

**DOI:** 10.1515/almed-2021-0082

**Published:** 2021-11-25

**Authors:** Guillermo Velasco de Cos, Isabel Sánchez-Molina Acosta, Marta Iturralde Ros, Ainhoa Maiztegi Azpitarte, Ana Moyano Martinez

**Affiliations:** Hospital Universitario Marqués de Valdecilla, Santander, Cantabria, Spain; Hospital Comarcal de Laredo, Laredo, Cantabria, Spain

**Keywords:** colestasis, enoxaparina, hepatitis, toxicidad, transaminasas

## Abstract

**Objectivos:**

El uso de heparina de bajo peso molecular está muy extendido en la práctica clínica. En muy raras ocasiones, algunos pacientes desarrollan hepatitis pocos días después de haber iniciado el tratamiento, y la inmediata suspensión del mismo resulta determinante a la hora de evitar que esta se cronifique.

**Caso clínico:**

Estos episodios suelen producirse solo en presencia de niveles elevados de transaminasas. Presentamos el caso de un paciente que presentó hepatotoxicidad con patrón colestásico, que es muy poco frecuente en este tipo de fármaco.

**Conclusiones:**

La hepatitis asociada al uso de heparinas es una entidad infradiagnosticada. Dado que esta patología no se suele manifestar con un claro perfil clínico, el laboratorio juega un papel fundamental en su diagnóstico.

## Introducción

La terapia anticoagulante con heparina no fraccionada (HNF) se lleva utilizando desde 1939. Sus efectos adversos más destacables son el riesgo de sangrado, la trombosis asociada a trombocitopenia y la trombocitopenia inmune. Hacia finales del siglo XX se empezaron a comercializar las HBPM (heparinas de bajo peso molecular), con menos efectos adversos que la HNF y con un ajuste de dosis más sencillo, ya que no requieren controles analíticos periódicos [1].

Entre los efectos adversos de las HBPM y la HNF destaca la aparición de hepatitis. El número de casos descritos es muy limitado, por lo que se desconoce su verdadera prevalencia. Conforme a las definiciones más recientes de hepatotoxicidad asociada a la heparina, esta se desarrolla cuando se produce un incremento tres veces por encima de límite superior del intervalo de referencia en el valor de las transaminasas, que no se pueda justificar por otras causas [2]. Se estima que la hepatotoxicidad se da en el 5% de los pacientes tratados con HNF y en entre el 5 y el 10% de los pacientes tratados con HBPM [3]. Entre estos últimos, la presentación varía dependiendo del fármaco. La Tinzaparina fue retirada del mercado en 2011 a causa de que el 13,3% de los pacientes desarrollaba este efecto adverso. Por otro lado, de las dos heparinas más utilizadas actualmente, la deltaparina es la que se asocia a una mayor incidencia de la hepatitis, existiendo muy pocos casos asociados al uso de la enoxaparina [4].

La hepatotoxicidad por HBPM se puede dividir en dos patrones: colestásico y hepatocelular, siendo el segundo más frecuente. El patrón hepatocelular muestra un aumento en los niveles de alanina aminotransferasa (ALT), con escasa elevación de la fosfatasa alcalina (FA) o la gamma-glutamil transferasa (GGT). Por otro lado, el patrón colestásico se define como la elevación de la fosfatasa alcalina dos veces por encima del límite superior del intervalo de referencia y una proporción ALT/ALP< 2. Este patrón se da en el 30% de los casos de hepatitis inducidas por fármacos, con una mayor supervivencia frente al patrón hepatocelular. En ambos patrones, las enzimas hepáticas se normalizan tras la suspensión del fármaco. No obstante, en el patrón colestásico, los niveles descienden más lentamente, existiendo una mayor probabilidad de cronificación.

De este modo, el diagnóstico temprano es esencial. La elevación de la bilirrubina, la fosfatasa alcalina y el tiempo de protrombina están asociadas a una mayor gravedad y riesgo de cronificación.

## Caso clínico

Presentamos el caso de un paciente de 83 años con diagnóstico de Parkinson y diabetes mellitus tipo 2 en tratamiento con ropinirol, carbidopa / levodopa y metformina / sitagliptina. El paciente no consumía alcohol, productos de herboristería o tabaco, ni tenía ningún otro hábito tóxico.

Actualmente, el paciente se encuentra en seguimiento por parte del Servicio de Hematología por una anemia arregenerativa no carencial, en el contexto de un síndrome linfoproliferativo crónico tratado con rituximab y con sangrado gastrointestinal no filiado. Se le realizó una laparotomía para determinar el origen del sangrado, tras el cual se le prescribió paracetamol y enoxaparina durante siete días.

Una semana después del procedimiento, el paciente acudió a la consulta, observándose una alteración aguda en los parámetros de función hepática, con: AST: 128 U/L [14–35 U/L], ALT: 177 U/L [10–49 U/L], GGT: 650 U/L [<73 U/L], ALP: 428 U/L [46–116 U/L], TSH: 1.57 mU/L [0.55–4.78], con bilirrubina total normal y sin síntomas asociados.

Con anterioridad a la intervención, y tras la administración de dos dosis de rituximab, mostró un perfil hepático inalterado, con serologías negativas y hemograma con una hemoglobina de: 8.1 g/dL [12–15.5 g/dL], hematocrito: 23.5% [34–46%].

A la luz de estos nuevos hallazgos analíticos, se decidió realizar una ecografía abdominal, además de solicitar serologías para hepatitis A, B, C, E y citomegavirus. La ecografía mostró un hígado de contornos lisos, con un parénquima ligeramente granular y residuos litiásicos en el interior de la vesícula. Los resultados negativos de las serologías para los diferentes virus descartaron un origen vírico de la infección. Se repitieron las serologías, obteniendo los mismos resultados. Los resultados negativos descartaron una etiología autoinmune.

Siete días después se volvió a realizar una analítica de control, que mostró niveles normalizados de transaminasas AST: 13 U/L, ALT: 54 U/L, con elevación persistente de GGT: 317 U/L y ALP: 218 U/L. La bilirrubina total permaneció en valores normales. Dos meses después del inicio del episodio, el paciente continúa con valores elevados de GGT y ALP: 132 U/L y 110 U/L, respectivamente. La evolución de los parámetros de función hepática analizados se muestra en la Tabla 1.

**Tabla 1:** Evolución de la función hepática.

	Estado basal	Día 1	Día 7	2º mes	3^er^ mes	7º mes
GGT (<73 U/L)	54	650	317	132	105	63
ALP (46–116 U/L)	87	428	218	110	104	95
AST (14–35 U/L)	13	128	13	17	20	18
ALT (10–49 U/L)	12	177	54	53	16	12
Bilirrubina total (0,2–1,1 mg/dL)	0.6	0.5	0.4	0.4	0.5	0.7

## Discusión

Se desconoce el mecanismo mediante el cual se produce la hepatotoxicidad asociada a la heparina. En los últimos años, se han publicado estudios que relacionan el daño hepático con el aumento de la proteína HMGB1, que las células liberan tras los procesos de necrosis, y actúa como señal de respuesta del sistema inmune innato. Se ha observado que, tras el segundo día de administración de la heparina, esta proteína aumenta, por lo que podría inducir toxicidad celular indirectamente [4]. Otros autores sugieren una citotoxicidad directa, dado que tras la administración de enoxaparina, se produce un aumento de los niveles séricos de miR-122, un biomarcador intracelular específico de células biliares [5].

Otro posible mecanismo es la alteración de la permeabilidad de la membrana del hepatocito. Sin embargo, este último mecanismo es poco frecuente en los fármacos metabolizados por desulfatación, como es el caso de la HBPM [6].

Para establecer un diagnóstico definitivo, es necesario descartar otras causas de daño hepático, como las infecciones virales, los hábitos tóxicos del paciente y la autoinmunidad. En este caso, el paciente no tenía hábitos tóxicos, y tanto la etiología vírica como autoinmune fueron descartadas, a raíz de los resultados analíticos negativos obtenidos.

La hepatotoxicidad parece estar relacionada con el sexo y edad del paciente, así como con la dosis del fármaco administrado. Los varones de mayor edad presentar mayor predisposición a desarrollar esta complicación [7]. Sin embargo, los estudios publicados hasta la fecha no son muy representativos, ya que se basan en pequeñas muestras de pacientes. En la mayoría de los casos, la alteración analítica del perfil hepático, comienza cinco días después de haber iniciado el tratamiento [8], alcanzando niveles máximos a los 7 días, para volver a valores normales 2 semanas después de la suspensión del tratamiento [9]. En nuestro caso, el inicio de la alteración en el perfil hepático y los valores máximos observados coinciden con los descritos en la literatura. Por otro lado, la recuperación a niveles normales llevó mucho más tiempo, lo que encajaría con la tendencia a la cronificación descrita en los casos de hepatotoxicidad colestásica.

Se estima que entre el 5 y el 13% de los pacientes con perfil colestásico desarrollan enfermedad hepática crónica, y entre el 5 y el 14% acaban falleciendo o precisando un trasplante. Los mejores factores predictores de mortalidad son las transaminasas y la bilirrubina. En este patrón, los valores de bilirrubina pueden variar, aunque suelen aparecer elevados con mayor frecuencia en el patrón hepatocelular, lo que se asocia a una mayor gravedad [6]. Unos niveles normales de bilirrubina y ligeramente elevados de transaminasas indican una menor gravedad de la alteración.

Entre las manifestaciones clínicas de la colestasis inducida por fármacos se incluyen ictericia, prurito, náuseas, malestar general y eosinofilia. Sin embargo, el perfil clínico de nuestro paciente era muy poco específico, por lo que el diagnóstico se basó en el perfil bioquímico.

Dado que no existen biomarcadores que permitan establecer un diagnóstico definitivo, el diagnóstico de este proceso se suele realizar por exclusión. La herramienta más utilizada actualmente es CIOMS/RUCAM (Método de Evaluación de la Causalidad del Consejo Internacional de Ciencias Médicas / Reoussel Uclaf) [4]. En esta escala, se trata de establecer la relación causal entre el fármaco responsable y el daño hepático. Se trata de un sistema de puntuación (Tabla 2) [6] que clasifica la sospecha como "definitiva o muy probable" (puntuación> 8), "probable" (puntuación 6–8), "posible" (puntuación 3–5), "improbable" (puntuación 1–2) y "se excluye" (puntuación <= 0). El caso que aquí presentamos obtuvo una puntuación de 6, lo que, según la escala, indica un diagnóstico probable de hepatotoxicidad inducida por heparinas de bajo peso molecular.

**Tabla 2:** Puntuación CIOMS/RUCAM para la evaluación de HTID Modificado a partir de: Danan G, y col [6].

CIOMS/RUCAM						
Tipo de daño hepático	Hepatocelular		Valor	Colestático/mixto		Valor
Criterios cronológicos	Primera exposición	Segunda exposición		Primera exposición	Segunda exposición	
Duración del tratamiento hasta el inicio de los síntomas	5–90 días	1–15 días	2	5–90 días	1–90 días	2
	<5 o >90 días	>15 días	1	<5 o >90 días	>90 días	1
Tiempo transcurrido desde la suspensión del tratamiento al inicio de los síntomas	>15 días	≥15 días	1	≤30 días	≤30 días	1
**Curso de la enfermedad**	**Diferencia entre el valor máximo de ALT y el límite superior normal**			**Diferencia entre el valor máximo de FA y el límite superior normal**		
En el momento de la suspensión de la medicación	>50% de mejora en 8 días >50% de mejora en 30 días Falta información o no se ha producido mejora Empeoramiento o mejora <50% en 30 días-		3 2 0 −1	>50% de mejora en 180 días <50% de mejora en 180 días Falta información o no se ha producido mejora		2 1 0
Factores de riesgo	Edad (≥55 años)		1	Edad (≥55 años)		1
	Consumo de alcohol		1	Consumo de alcohol o embarazo		1
Terapia concomitante	Ninguna o se desconoce		0	Ninguna o se desconoce		0
	Posible fármaco implicado		−1	Posible fármaco implicado		−1
	Hepatotoxina conocida, posible implicación		−2	Hepatotoxina conocida, posible implicación		−2
	Efecto probado en estos casos		−3	Efecto probado en estos casos		−3
	No se dispone de información		0	No se dispone de información		0
Exclusión de otras causas no farmacológicas	Descartado		2	Descartado		2
	Causas posibles no investigadas		entre -2 y 1	Posible pero no confirmado		entre -2 y 1
	Otra causa probable		−3	Otra causa probable		−3
Información de hepatotoxicidad previa	Reacción desconocida		2	Reacción desconocida		2
	Existen casos publicados, pero no incluido en ficha técnica		entre -2 y 1	Existen casos publicados, pero no incluido en ficha técnica		entre -2 y 1
	Incluido en la ficha técnica		−3	Incluido en la ficha técnica del fármaco		−3
Respuesta a la readministración del fármaco	Positivo		3	Positivo		3
	Compatible		1	Compatible		1
	Negativo		−2	Negativo		−2
	No disponible o no interpretable		0	No disponible o no interpretable		0
	Concentraciones plasmáticas tóxicas		3	Concentraciones plasmáticas tóxicas		3
Pruebas de laboratorio validadas, con buen valor predictivo	Positivos		3	Positivo		3
	Negativos		−3	Negativos		−3
	No disponible o no interpretable		0	No disponible o no interpretable		0

CIOMS, Consejo Internacional de Ciencias Médicas; RUCAM, Reoussel Uclaf Método de Evaluación de la Causalidad; ALT, alanina aminotransferasa; FA, fosfatasa alcalina.

Por otro lado, existen unos criterios analíticos/bioquímicos para la suspensión del tratamiento con HBPM debido al riesgo de hepatotoxicidad. La mayoría se basan en un incremento de los valores de AST tres veces por encima del límite superior del intervalo de referencia, asociados a otra alteración bioquímica, como un incremento en los valores de bilirrubina o una alteración en la INR [10]. La elevación aislada de las transaminasas cuando este incremento se prolonga durante más de dos semanas es también un criterio de suspensión de tratamiento.

En el caso de la HBPM, el tratamiento ya se suele haber suspendido en el momento del diagnóstico, como fue nuestro caso. En los pacientes en los que se logra un diagnóstico temprano, se recomienda sustituir el tratamiento por fondaparinux, un inhibidor sintético selectivo del factor Xa, que actúa potenciando el efecto de la antitrombina III. Existe evidencia de que la sustitución con fondaparinux no interfiere en la reducción de las enzimas hepáticas [4].

Es importante tener en cuenta esta causa de hepatotoxicidad, para lograr un diagnóstico temprano basado en el uso de escalas adecuadas, evitando así someter a los pacientes a estudios diagnósticos invasivos innecesarios.

## Lecciones aprendidas

– La hepatitis inducida por la heparina es un efecto adverso poco frecuente infradiagnosticado.
– El laboratorio resulta esencial a la hora de obtener un diagnóstico temprano, ya que la mayoría de los pacientes al principio no presentan manifestaciones clínicas.
– Existen dos patrones: hepatotóxico, el más común, y colestásico, que es menos frecuente.
– La suspensión del tratamiento permite la recuperación del paciente, normalmente 14 días después de la misma. En el patrón colestásico, las alteraciones bioquímicas tardan más en desaparecer.
– La hepatitis es más frecuente en pacientes tratados con HBPM que en aquellos tratados con HNF, siendo fondaparinux la principal alternativa terapéutica.
